# Geographic Variation in the Status Signals of *Polistes dominulus* Paper Wasps

**DOI:** 10.1371/journal.pone.0028173

**Published:** 2011-12-09

**Authors:** Elizabeth A. Tibbetts, Oksana Skaldina, Vera Zhao, Amy L. Toth, Maksim Skaldin, Laura Beani, James Dale

**Affiliations:** 1 Ecology and Evolutionary Biology, University of Michigan, Ann Arbor, Michigan, United States of America; 2 Department of Nature Protection Nikitsky Botanical Garden, National Scientific Center Nikita, Yalta, Crimea, Ukraine; 3 Department of Ecology, Evolution, and Organismal Biology and Department of Entomology, Iowa State University, Ames, Iowa, United States of America; 4 Joint Biotechnology Laboratory, Department of Chemistry, Mathematics and Natural Sciences Faculty, University of Turku, BioCity 6A, Turku, Finland; 5 Department of Evolutionary Biology “Leo Pardi”, University of Florence, Firenze, Italia; 6 Institute of Natural Sciences, Albany Campus, Massey University, Auckland, New Zealand; Cajal Institute, Consejo Superior de Investigaciones Científicas, Spain

## Abstract

Understanding intraspecific geographic variation in animal signals poses a challenging evolutionary problem. Studies addressing geographic variation typically focus on signals used in mate-choice, however, geographic variation in intrasexual signals involved in competition is also known to occur. In *Polistes dominulus* paper wasps, females have black facial spots that signal dominance: individuals wasps with more complex ‘broken’ facial patterns are better fighters and are avoided by rivals. Recent work suggests there is dramatic geographic variation in these visual signals of quality, though this variation has not been explicitly described or quantified. Here, we analyze variation in *P. dominulus* signals across six populations and explore how environmental conditions may account for this variation. Overall, we found substantial variation in facial pattern brokenness across populations and castes. Workers have less broken facial patterns than gynes and queens, which have similar facial patterns. Strepsipteran parasitism, body size and temperature are all correlated with the facial pattern variation, suggesting that developmental plasticity likely plays a key role in this variation. First, the extent of parasitism varies across populations and parasitized individuals have lower facial pattern brokenness than unparasitized individuals. Second, there is substantial variation in body size across populations and a weak but significant relationship between facial pattern brokenness and body size. Wasps from populations with smaller body size (e.g. Italy) tend to have less broken facial patterns than wasps from populations with larger body size (e.g. New York, USA). Third, there is an apparent association between facial patterns and climate, with wasp from cooler locations tending to have higher facial pattern brokenness than wasps from warmer locations. Additional experimental work testing the causes and consequences of facial pattern variation will be important, as geographic variation in signals has important consequences for the evolution of communication systems and social behavior.

## Introduction

There has been growing interest in the causes and consequences of geographic variation in signals [1], as many species exhibit substantial phenotypic and genotypic variation across populations. Well known examples include bird species which have song dialects that vary dramatically across only a few miles [2], [3], and guppies that show dramatic geographic variation in the extent of orange and black body coloration [4]. Because selection and drift typically reduce variation, understanding the causes and consequences of variation in natural populations is an important goal of evolutionary biology [5], [6], [7], [8].

Geographic variation in signals may arise via genotypic differences between populations or in response to environmentally-based variation. First, in many instances signal variation is a consequence of genetic divergence between populations. For example, social factors such as the degree and nature of interspecific and intraspecific competition shape signal evolution such that signals can diverge genetically in sympatry in order to reduce the costs of inter-specific mating or agonistic competition with heterospecifics [9], [10], [11]. Genetic divergence that arises via non-selective processes such as genetic drift may also produce geographic variation in signals [12]. Second, geographic variation in signals may also be a consequence of developmental plasticity that reflects variation in the environment. For example, call frequency in anurans typically co-varies with body size, so populations with different average body sizes also have different call frequencies [13]. Similarly, song dialects can quickly arise when young males learn their song from neighboring males and populations are philopatric [14].

Here, we explore geographic variation in signaling within *Polistes dominulus* paper wasps, and test whether environmental variation plays a role in the variation. Previous work in the northern United States has shown that *P. dominulus* facial patterns function as a conventional signal of agonistic ability. Wasps with more broken, wavy black facial spots are better able to dominate rivals than wasps with less broken, wavy facial spots [15], [16], [17]. Further, wasps use facial pattern brokenness to assess the agonistic ability of unfamiliar rivals prior to interacting [18], [19]. However, subsequent work on *P. dominulus* in other populations suggests there is dramatic geographic variation in *P. dominulus* facial patterns. For example, a large fraction of the *P. dominulus* queens in Spanish and Italian populations have identical, yellow faces that lack any black spots [20], [21]. The surprisingly low facial pattern variation within these *P. dominulus* raises questions about 1) the extent of facial pattern variation across populations and 2) the causes of this variation.

Here, we describe facial pattern variation across six *P. dominulus* populations. Previous work has shown that caste influences facial patterns [22], likely because workers are smaller and in poorer physical condition than queens [23]. Therefore, we start by testing how caste and geographic origin influence facial pattern variation. Then, we explore whether developmental plasticity between populations may play a role in driving geographic variation. Previous work has shown that facial pattern development is condition-dependent [24], [25] and correlated with body size [15], [22] but see [21], [24]. Therefore, we examine whether geographic variation in facial patterns is associated with geographic variation in body size and strepsipteran parasitism that is thought to influence host development and phenotype [26], [27], [28]. We also explore how facial pattern variation is associated with geographic variation in climate, as climate is well known to influence invertebrate size and condition [29].

## Methods

### Collection methods

In this long-term study across populations, morphological measurements, as well as caste/parasitism evaluation, were obtained from each collection through different but comparable methods (Table 1). No special permits were required for the collections, as *P. dominulus* are not protected or endangered. Pictures from each population are included in a video in the supplemental material (Movie S1).

**Table 1:** Methods in each population.

Population	Caste	Facial pattern	Size	Strepsipteran parasitism	Average High/Low temperature in May; January(C)
Michigan, USA	Collection date	Photographs	Head width	No parasites in USA. Scored as unparasitized.	20/8 ; −1/−8
New York, USA	Collection date	Photographs	Head width	No parasites in USA. Scored as unparasitized.	19/6; −1/−10
Hungary	Dissection and collection date	Photographs	Head width	Dissection	20/12; 1/−2
Italy	Dissection and collection date	Photographs	Head width	Dissection	23/11; 9/1
Kherson, Ukraine	Collection date	Drawings	Not available	Not available	21/10; 0/−4
Yalta, Ukraine	Collection date	Drawings	Not available	Not available	18/12; 6/2

Average temperature during May and January acquired from www.weatherreports.com for Ann Arbor MI, Ithaca NY, Veszorem Hungary, Florence Italy, Kherson Ukraine, and Yalta Ukraine.

### Michigan, USA

*P. dominulus* foundresses were collected from sites within 15 km of Ann Arbor, MI, USA in early May 2008. Nests containing nest-founding queens were collected in May, approximately 2 weeks after nest foundation. Nests containing offspring were collected from similar sites in early August. The non-egg laying individuals currently on the nest were considered workers. Individuals that eclosed from these same nests in mid to late August, after males, were considered gynes. The Michigan sample contained 854 wasps.

### New York, USA

*384 P. dominulus* foundresses were collected from sites within 10 km of Ithaca, NY, USA between May 14 and May 19 2001 approximately 2 weeks after nest foundation.

### Italy

132 wasps were collected in Tuscany, Italy in two different years as part of another study on parasitism by *Xenos vesparum*. In March 2008, aggregating overwintering gynes, both healthy and parasitized, were collected. In summer 2008, three nests were infected in the lab (Florence) with *X. vesparum* triungulins and the first offspring (i.e. workers) were collected; additional pre-overwintering gynes were collected in September 2008. In March 2009, aggregating overwintering gynes were collected. In June 2009, 9 worker phase nests were collected in Florence and maintained in the laboratory until the emergence of workers. Some nests were naturally or artificially infected and others were uninfected. In August 2009, queens, workers and aggregating gynes were collected.

### Hungary

42 wasps were collected from worker phases nests near Lake Balaton, Transdanubian Region, Hungary in June and July 2009 as part of aforementioned study on parasitism by *X. vesparum*.

### Kherson, Ukraine

Wasps were studied at the Black Sea Biospherical Reserve which is located on the north coast of the Black Sea near the Tendrovskiy and Yagorlizkiy gulfs. Foundresses were individually marked approximately one month after nest foundation in early May 2003 and 2004. Nests were censused twice a week throughout the rest of the colony cycles. Females that eclosed before males were considered workers, while later eclosing females were considered gynes. The Kherson sample contained 1058 wasps. All individuals from Ukraine were not dissected to measure parasitism, so they are not included in the parasitism analysis. Permission for this research was obtained from the director of the Reserve Dr. Dmitriy Chernyakov.

### Yalta,Ukraine

Wasps were studied in the Nikitsky Botanical Garden in Yalta, Ukraine. Wasps were collected and photographed at the end of July. The Yalta sample contained 58 gynes. All individuals were not dissected to measure parasitism, so they are not included in the parasitism analysis. Permission for this research was obtained from from the deputy director, Dr. Gennadiy Zakharenko.

### Caste

In Michigan and Ukrainian wasps, caste was identified by collection date (Table 1), as described above. In Hungarian and Italian wasps, caste determination was more difficult, as part of the sample came from aggregations and the *Xenos* parasite influences host phenotype (body size, ovarian development, and fat body [27]). As a result, multiple sources of information were used for caste determination. Each individual was dissected and caste was determined using ovarian development, fat body [23], [27], date of wasp emergence (when available), and date of aggregation collection. For example, in hibernation aggregations, parasitized wasps are larger and have more fat bodies than individuals in fall aggregations. Therefore, the former are likely to be putative gynes (last offspring), while the latter may also include workers (first offspring).

### Size

Head width at the widest part of the head was used as the measure of body size (Table 1). In Italian and Hungarian populations, head width was measured with digital microcalipers and was accurate to the nearest 0.01 mm. In Michigan and New York wasps, head width was measured from head photographs taken with a dissecting scope and containing a size standard. Size information was collected for all wasps from New York and a subset of wasps from Michigan, Italy, and Hungary.

### Parasitism

The abdomens of all wasps from Italy and Hungary were dissected in order to verify caste differences (queens with large ovaries, workers with small ovaries, and gynes with large fat bodies and small ovaries) and the number, stage, and sex of all *Xenos* parasites was noted as in Beani (2006). These data were useful for caste assignment, but were inadequate for posthoc analysis of how parasites influence brokenness. Strepsiptera do not parasitize *P. dominulus* in the United States population [30], so all wasps from Michigan and New York were considered unparasitized. Data on parasitism are lacking from Ukraine populations (see above).

### Facial pattern analysis

**Figure 1:**
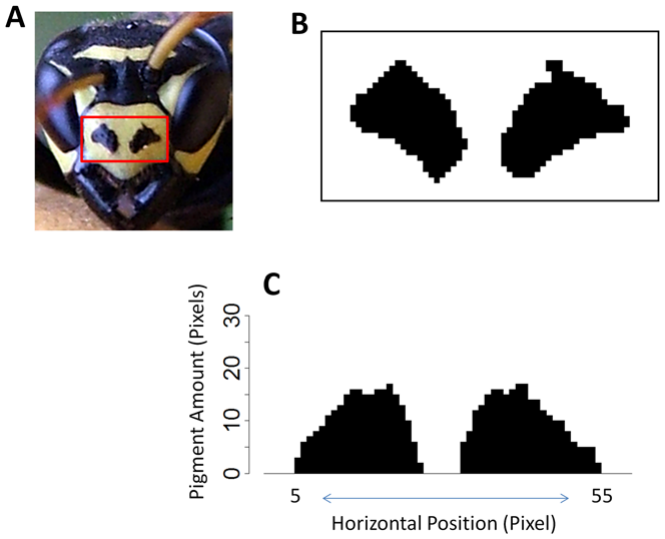
Illustration of the method used for analyzing facial pattern brokenness. A) *P. dominulus* with a red box around the facial area that is analyzed for brokenness, B) 30×60 pixel bitmap of the facial pattern variation, C) The number of pixels of black pigment within each vertical column along the horizontal length of the clypeus is counted and the standard deviation from column 5 to 55 is calculated.

The signal phenotype in *Polistes dominulus* was assessed by analyzing a digital picture of each wasp face with Adobe Photoshop (Fig. 1). A wasp's facial pattern “brokenness” is the best predictor of dominance and takes into account the number, size, and shape of black spots on the center of the wasp's clypeus [15], [19], [24]. Brokenness was determined by converting the area of the clypeus containing the population-wide badge variability into a 30×60-pixel bitmap. Then, the number of black pixels within each vertical column along the horizontal length of the clypeus was counted. The total disruption or brokenness of the black facial pattern is the key parameter, so the standard deviation of the black pigment from pixels 5 to 55 along the horizontal gradient of the clypeus was calculated. The first and last five pixels were excluded from the brokenness analysis because the edges of the clypeus are black. As a result, wasps with black in the first and last five pixels of the clypeus actually have facial patterns that appear somewhat less broken. Facial patterns with more, wavier spots are higher on the brokenness index than facial patterns with fewer, smoother spots. Wasps with no black on their clypeus are scored as having a brokenness of zero.

The facial pattern of wasps from Kherson were originally scored using a categorical scoring scheme (Fig. S1). Photographs were not available, so we translated the categorical scores into brokenness by measuring the brokenness of each drawing. We tested whether this method produced reliable brokenness measures by categorizing 49 pictures using the drawings, then analyzing the brokenness of the same pictures using the pattern analysis method described above. The brokenness of the pictures and the brokenness assigned using the drawings were extremely similar (F_1,48_ = 53.3, p<0.001, r^2^ = 0.52). Therefore, categorization method provides a reliable way to analyze facial patterns when a digital camera is unavailable.

### Statistical analysis

Brokenness scores were log + 1 transformed prior to analyses to improve normality. Statistical analyses were performed using mixed linear models that included nest of origin as a random effect to account for potential similarity within individuals collected from the same nest. In Italy and Hungary, some individuals were collected from pre-hibernation and hibernating aggregations, so aggregation rather than nest was included as the random effect. Non-significant interactions were removed from the final models, but are reported below.

## Results

### General survey of facial pattern in relation to caste differences and geographic variation

**Figure 2:**
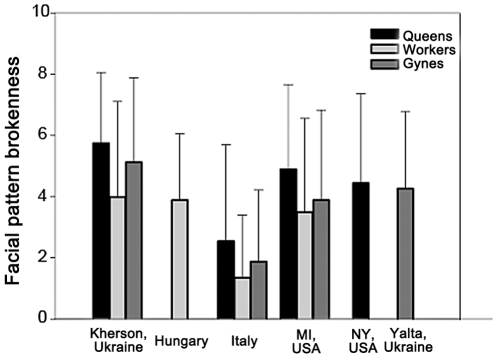
Mean (± SE) facial pattern brokenness of *P. dominulus* workers, gynes, and queens in six different populations.

**Figure 3:**
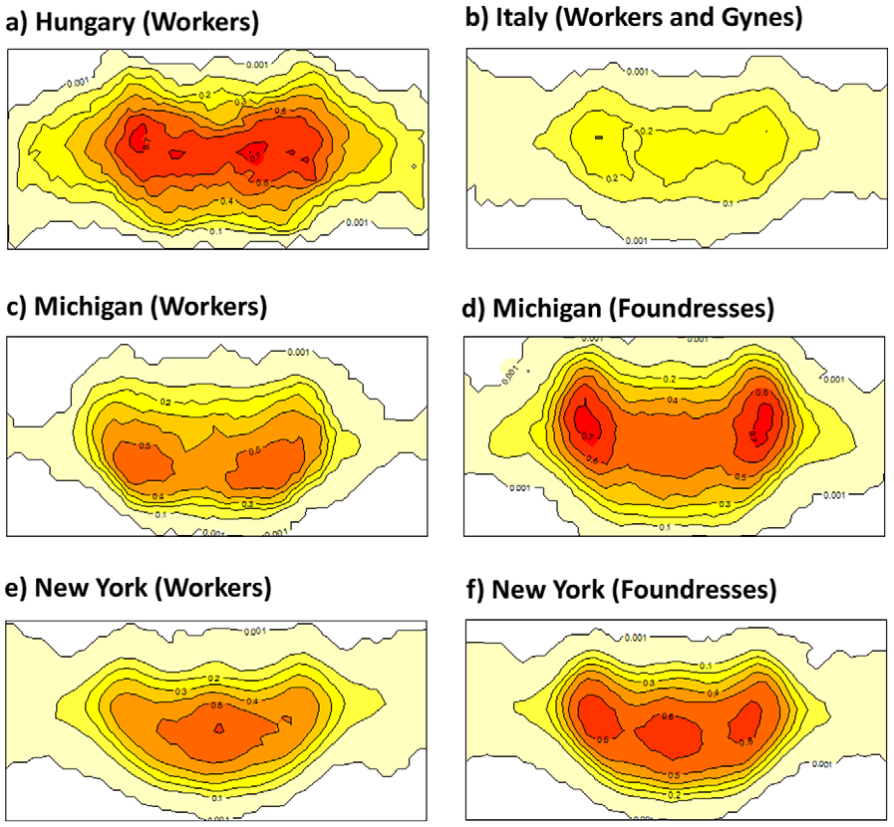
Contour maps of population-level clypeal melanin pigmentation in different groups of wasps. Each contour line represents the percentage of wasps in the population with pigment present in that region of the clypeus. These maps demonstrate that in general pigmentation is most common in central area of the face, that different locations have dramatically different population level patterns of pigmentation, and that workers tend to have less complex patterns than foundresses. Number of photographs used to create the contour maps: a) 42, b) 132, c) 87, d) 147), e) 425), f) 384.

Facial pattern brokenness was influenced by caste (Fig. 2, F_2,1711_ = 40.3, p<0.0001) and population (F_5,370_ = 4.41, p = 0.001, see Fig. 3). There was no significant interaction between caste and population (F_5,1404_ = 0.47, p = 0.80), indicating that the relationship between facial pattern and caste was similar across populations. LSD posthoc analysis illustrates that workers had the lowest facial pattern brokenness (worker vs. gyne p<0.0001, worker vs. queen p<0.0001), while gynes and queens have facial patterns with similarly high facial pattern brokenness (gyne vs. queen p = 0.17). Workers tend to have less complex black patterns on the clypeus (Fig. 3C and 3E) than foundresses or gynes (Fig. 2D and 2F).

Regardless of caste, wasps from Italy had the lowest facial pattern brokenness and differed from all other populations (p<0.05). Wasps from New York and Yalta had slightly more broken facial patterns (New York vs. Yalta P = 0.74, New York vs. Michigan p = 0.06, NY vs. all other p<0.05), though because of the small sample size in Yalta, Yalta only differed significantly from Italy (all other population comparisons p>0.05). Wasps from Michigan, Kherson, and Hungary had facial patterns with the highest brokenness. These three populations did not differ from each other or the Yalta population (all p>0.05), but generally differed from the other three populations (p<0.05 except MI vs. NY p = 0.06).

### Facial pattern brokenness and environmental traits

**Figure 4:**
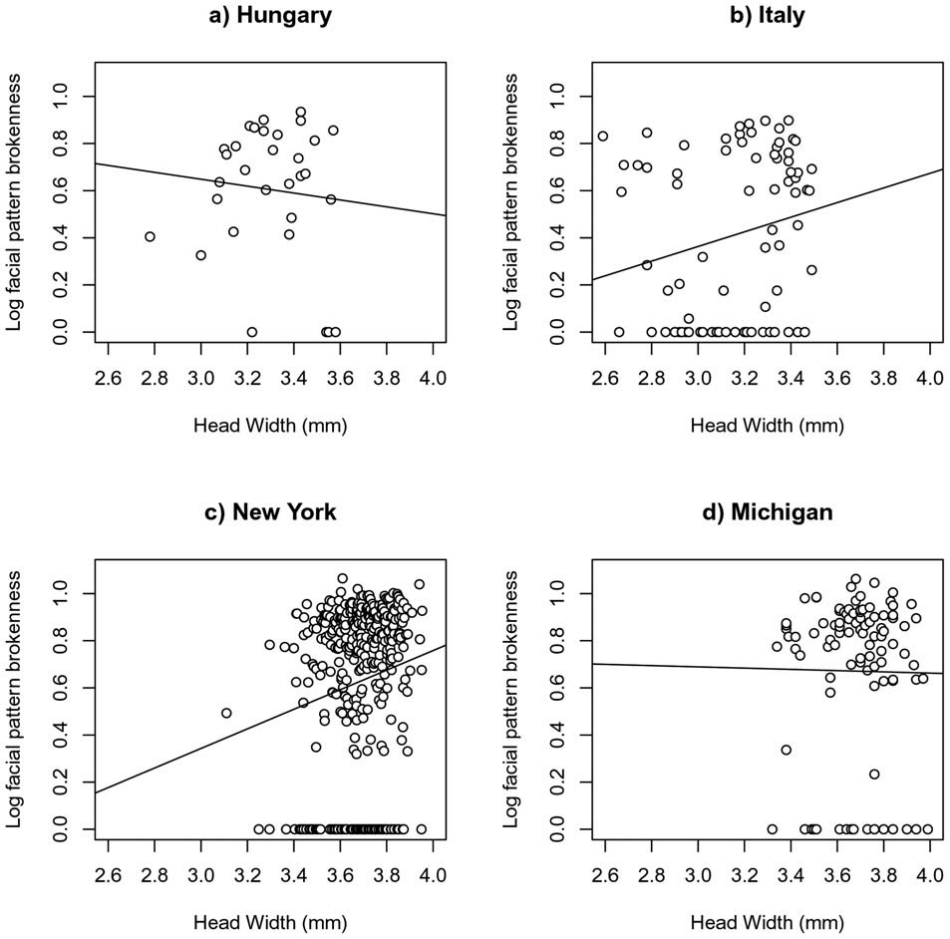
Relationship between facial pattern brokenness and body size in four populations. There is a similar, positive relationship between body size in Italy and New York, USA, but no relationship between body size and facial pattern brokenness in Michigan, USA and Hungary.

**Figure 5:**
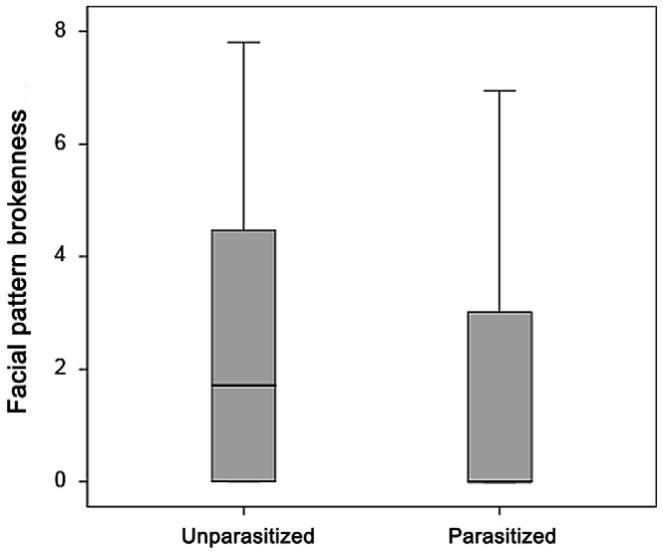
Facial pattern brokenness of parasitized vs. not-parasitized *P. dominulus* in the Italian and Hungarian populations. Parasitism is associated with lower facial pattern brokenness. Box indicates the 25^th^, 50^th^, and 75^th^ percentile, while the whiskers reflect the 95% confidence interval.

Body size and parasitism information was available in a subset of wasps in Michigan, New York, Italy, and Hungary. Within this subset, facial pattern was associated with population (Fig. 4, F_3,562_ = 2.7, p = 0.046), parasitism (Fig. 5, F_1,357_ = 7.35, p = 0.007), and the interaction between population and head width (Fig. 4, F_3,568_ = 2.5, p = 0.05). Caste (F_2,275_ = 0.82, p = 0.44), and body size alone (F_1,578_ = 0.45, p = 0.83) were not associated with facial patterns. None of the other two-way interactions influenced facial pattern brokenness (all p>0.25). Posthoc LSD analysis shows no significant differences between any two populations, though Michigan tends to have relatively higher facial pattern brokenness than New York (p = 0.07). All other comparisons p>0.50.

To ensure that the relationship between parasitism and facial pattern brokenness was not influenced by the inclusion of US populations that lack parasites, a separate analysis was performed in Italian and Hungarian wasps alone. Within these two populations, facial patterns were associated with parasitism (F_1,102_ = 6.4, p = 0.013), population (F_1,102_ = 10.2, p = 002), but not body size (F_1,102_ = 0.028, p = 0.87). Caste was not significantly associated with facial pattern (F_1,102_ = 2.4, p = 0.094), likely because of low statistical power. Gynes were primarily collected from two aggregations and collection location is included as random effects in the model. None of the two-way interactions influenced facial pattern brokenness (all p>0.25).

Given the relationships between body size and facial pattern brokenness across populations, a separate analysis was performed to test how body size alone varied across populations. Head width varied significantly across the four populations (F_3,231_ = 185, p<0.001). LSD posthoc analysis shows that Michigan and New York had the largest wasps (3.68 mm; Michigan vs. New York p = 0.089, Michigan or New York vs. Hungary or Italy p<0.001), Hungarian wasps were intermediate sized (3.27 mm; Hungary vs. Italy p<0.001), and Italian wasps had the smallest body size (3.05 mm).

## Discussion

There is substantial variation in *P. dominulus* facial patterns across populations and castes. In general, workers have less broken facial patterns than gynes or queens, while gynes and queens have similar facial pattern brokenness. The consistent relationship between caste and facial pattern across populations suggests that similar mechanisms underlie signal development across populations. We also found striking variation in facial patterns across populations, with Italian wasps having the lowest facial pattern brokenness and Kherson, Ukraine wasps having the highest facial pattern brokenness. Although additional research will be important to explicitly test the basis of this variation, our results suggest that ecological factors such as the influence of strepsipteran parasitism and climatic conditions on wasp development, may all play a role in the geographic variation in facial patterns.

The reduced facial pattern brokenness in workers relative to gynes and queens makes sense considering previous work on facial pattern development and paper wasp life history. Facial pattern development is influenced by larval nutrition and facial patterns are correlated with body size [22], [24], [25]. As workers are typically smaller and in poorer nutritional condition than gynes and queens [23], their low facial pattern brokenness is not surprising. Our results match previous work in New York and Michigan populations that found lower facial pattern brokenness in workers than gynes [22], [24]. There have been no previous comparisons of gyne and queen facial patterns. However, gynes are theoretically expected to have facial patterns that are similar to foundresses, as gynes overwinter to become queens the following year [31].

Our results suggest that much of the geographic variation in facial patterns may be a consequence of developmental plasticity linked to geographic variation in the average condition (e.g. larval nutrition and health) of *P. dominulus*. Facial pattern development is condition-dependent [24], [25] and associated with body size [15], [22]. Therefore, factors that influence average body size or condition within a population also influence average facial pattern brokenness.

### Body size

Geographic variation in body size is associated with some the variation of *P. dominulus* facial patterns, as body size variation parallels facial pattern variation. For example, Italian wasps have the smallest body size and the lowest facial pattern brokenness, while New York wasps have larger body sizes and higher facial pattern brokenness. Interestingly, New York and Italian wasps fall along different ends of the same regression line, suggesting that different facial pattern brokenness in the populations could be a developmental by-product of selection for different body size in the two populations. Of course, body size accounts for only a fraction of the variation in facial pattern brokenness across populations, as the relationship between size and facial pattern is weak and inconsistent. Therefore, size may not directly modulate facial pattern development. Instead, factors such as food availability during larval development [24] or climate may influence both body size and facial patterns. If the same factor influences both body size and facial patterns, it may commonly produce a relationship between these variables, even if body size and facial patterns are not causally linked.

### Parasitism

Strepsipteran parasitism likely plays a small but significant role in facial pattern variation, as parasitism affects host phenotype and reduces facial pattern brokenness. In addition, levels of parasitism vary dramatically across the geographic range of *P. dominulus*. For example, Italy has high levels of parasitism and low facial pattern brokenness, while there are no strepsipteran parasites of *P. dominulus* in the US populations which have relatively high facial pattern brokenness. *P. dominulus* facial patterns are influenced by larval feeding [24], [25], so strepiptera likely reduce host facial pattern brokenness via nutritional costs imposed during development. The precise nutritional cost of strepisipteran parasitism remains untested, though the cost appears to be sustainable during larval stages [26]. There is strong, consistent evidence that strepsiptera influence host development, as stylopized wasps are smaller than healthy ones [21], [32], show higher levels of fluctuating asymmetry, and have less fat body depending on parasite load and sex [27]. Our results suggest that strepsiptera impose a sufficient nutritional burden to reduce the advertised quality of the host, in agreement with their lack of involvement in the colony hierarchy, social life [26], and dominance interactions in winter aggregations [33]. The relationship between adult advertised quality and parasitism could also be due to strepsiptera preferential parasitizing larvae in poor condition. However, this alternative is unlikely, as strepsiptera parasitize all the larval stages [26], without any selective host-seeking behavior [34]. Nevertheless, future experiments will be useful to confirm that parasitism itself reduces facial pattern brokenness.

A previous study failed to find an association between strepsipteran parasitism and *P. dominulus* facial patterns [21]. However, this study assessed the effect of parasitism not on brokenness but on the amount of facial pattern pigmented black and the number of black spots. Cervo et al. also used separate analyses to test the effect of parasitism on body size and facial pattern. Incorporating both size and facial pattern into a single analysis provides a more robust test than independent analyses. Moreover, our inter-population sample was larger and included both first and last wasp offspring, i.e. putative workers and gynes, two factors that may increase the power to detect the effect of parasitism on facial patterns.

### Climate

Although we do not have sufficient data to statistically test the role of climate in geographic variation, climate seems to co-vary with facial pattern variation. Populations from cooler locations, such as Michigan, tend to have more broken facial patterns than populations from warmer locations, such as Italy. Most strikingly, the two Ukrainian populations show differences in facial pattern that match temperature differences, though the populations are only about 300 km apart. Yalta has a subtropical climate that substantially warmer the winter than continental climate of Kherson (Table 1). The Yalta population also has lower facial pattern brokenness than Kherson. In addition, the population of *P. dominulus* with the lowest known facial pattern brokenness is in Conil de la Frontera, Spain which has a moderate climate and warm winters that rarely drop below freezing (average January low is 9°C) [20].

What accounts for the potential link between climate and facial pattern brokenness? Many species show temperature-based clines in size or condition and there has been extensive empirical and theoretical work on the basis of this variation [29], [35]. One important hypothesis for these clines is that the risk of starvation in harsh or unpredictable environments favors larger individuals in good condition, while milder environments favor smaller individuals in poorer condition [36], [37]. In *P. dominulus*, large body size and highly broken facial patterns are associated with increased survival under restricted food availability [38], though more work is needed to test how these factors influence overwinter survival [21]. Foundresses and workers control food availability, so they can potentially manipulate the size and condition of all offspring [31]. In fact, *P. gallicus* (ex *foederatus*) females emerging from laboratory colonies exposed to low temperatures had larger body sizes and better developed fat bodies [39]. Further, climate influences individual fat content in *Polistes biglumis*, as *P. biglumis* from colder populations have more abundant fat bodies than *P. biglumis* from warmer populations [40]. Therefore, selection for fewer, better offspring in harsh climates may be a primary factor driving the geographic variation in facial patterns. More explicit future analyses are important to further test this hypothesis.

### Other sources of variation: genetics

It is important to note that genotypic differences between populations, caused by local adaptation or drift, could also account for some of the geographic variation in facial patterns. A previous study using lab-reared *P. dominulus* found inconsistent heritability of facial pattern brokenness [24]. However mother daughter comparison in both the Michigan and Kherson populations demonstrate a positive relationship (EA Tibbetts, O Skaldina, M Skaldin unpublished data).

Much of the research on local adaptation in signaling has focused on selection to improve signal transmission as well as co-evolution with heterospecifics. Although selection to optimize signal transmission is unlikely to explain the variation in *P. dominulus* facial patterns, co-evolution with heterospecifics may play a role. *P. dominulus* have a flexible social life that involves both cooperative nesting and intense aggressive conflict with conspecifics [41], so individuals may be favored to look as distinct from heterospecifics as possible. *P. dominulus* also overlap with multiple species across their range and some of these species have variable facial patterns that may be involved in social signaling [42]. For example, *P. sulcifer* is a social parasite that usurps established *P. dominulus* nests and uses *P. dominulus* workers to rear her own offspring [43]. *P. sulcifer* also have variable facial patterns that are darker than *P. dominulus* facial patterns [44], so *P. dominulus* may be favored to have yellower facial patterns in areas of overlap with *P. sulcifer*. Additional work explicitly comparing facial patterns in populations with and without heterospecifics will be useful to assess whether co-evolution with heterospecifics plays a role in geographic variation.

### Consequences of variation

The variation in facial patterns across populations likely has interesting consequences for social signaling. In populations full of low quality individuals with entirely yellow faces, there is less facial pattern variation. For example, in Italy and Spain, over 50% of the population has identical, yellow facial patterns [20]. Reduced facial pattern variation may reduce the importance of facial patterns during social interactions. Experimental work suggests facial patterns play a larger role during rival assessment in Michigan than in Spain [18], [19], [45]. However, there is some evidence that facial patterns in the Spanish population provide useful information about fighting ability, as the proportion of the clypeus pigmented black is weakly correlated with dominance in Spain [20]. Although proportion of the clypeus pigmented black is a weak proxy for brokenness in most populations, it is likely to provide a decent estimate of advertise quality in Spain, when less than 10% of multiple foundresses nests have more than one queen with black facial pigment. One previous study [21] examined facial patterns in Italian *P. dominulus*, finding no relationship between facial patterns and dominance in a population with a yellow clypeus predominance (60%). However, a more complex, paired statistical model, such as the analysis performed by [20], may have yielded different results. Future work testing the signal value of *P. dominulus* facial patterns across populations will be important to test whether the importance of facial patterns in social signaling co-varies with the amount of facial pattern variation in a population.

### Conclusion

Overall, our results suggest that facial pattern variation across the geographic range of *P. dominulus* reflects developmental plasticity. Factors including strepsipteran parasitism, local climate, and food availability may influence the size and condition of local wasps. Facial patterns co-vary with these parameters such that populations that are heavily parasitized, in warm locations, or have small wasps will have lower facial pattern brokenness than other populations. Facial patterns play a significant role in social communication, so future experimental work examining the causes and social consequences of facial pattern variation will be useful.

## Supporting Information

Figure S1 **Drawing used to score the variation in** ***P. dominulus*** **facial patterns within the Ukrainian populations.**
(TIF)

Movie S1 **Video containing the bitmaps used to create Fig. 3, as well as pictures of *P. dominulus* from Italy, Hungary, Michigan, and New York.** Wasps with entirely yellow faces were typically not photographed.
(WMV)
