# Supplementary figures and images for: Geographic Variation in the Status Signals of Polistes dominulus Paper Wasps
Source: PLoS One. 2011 Dec 9;6(12):e28173. doi: 10.1371/journal.pone.0028173 (PMC3235107; doi:10.1371/journal.pone.0028173)

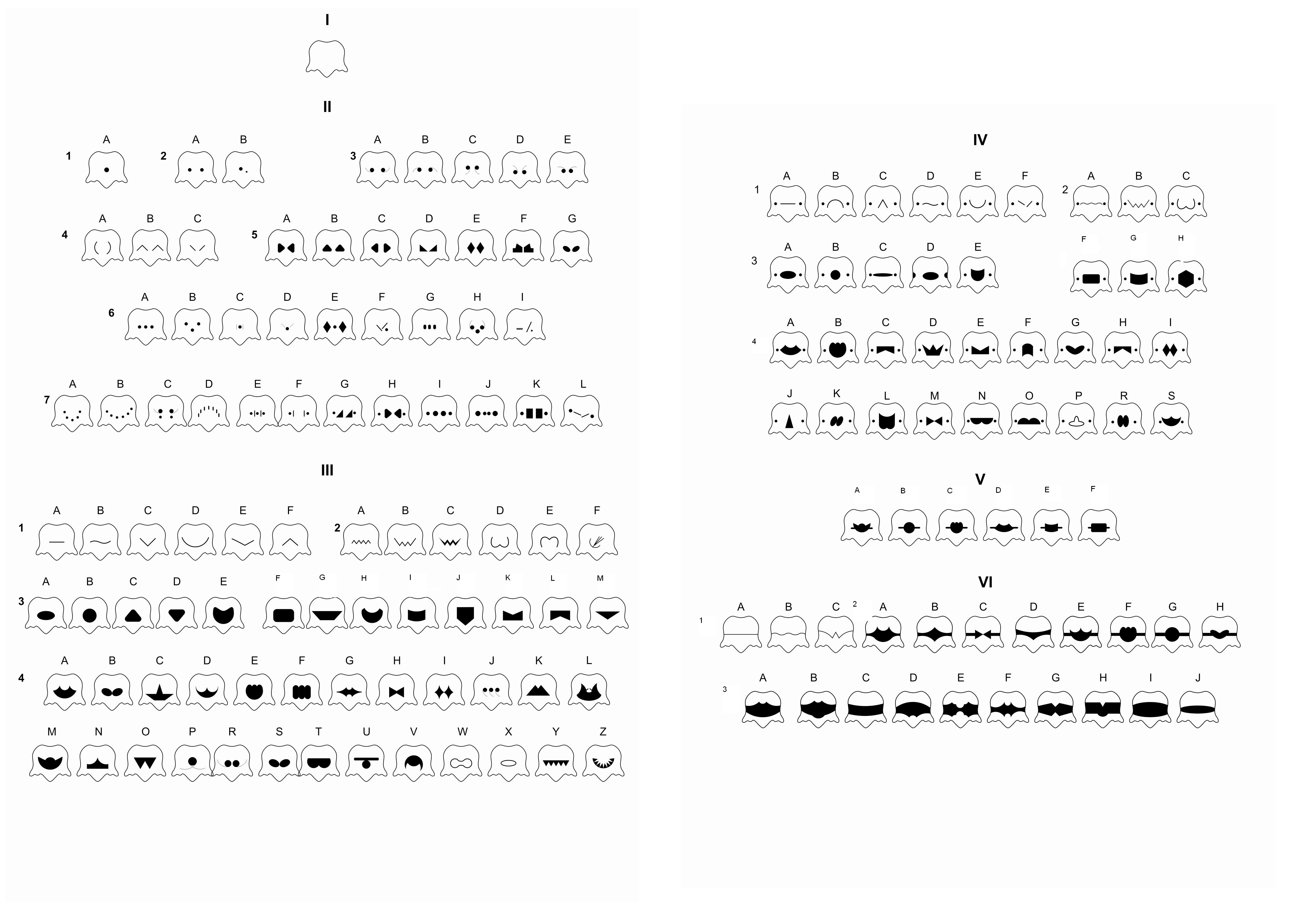

Supplement: Figure S1 — Drawing used to score the variation in P. dominulus facial patterns within the Ukrainian populations. (TIF) [file pone.0028173.s001.tif]
